# Design and cost-effectiveness assessment of a model based on point-of-care testing for the improvement of the resolution of hospital emergencies

**DOI:** 10.1515/almed-2024-0210

**Published:** 2025-07-18

**Authors:** Marta Jimenez-Barragan, Antonio Leon-Justel, Catalina Sanchez-Mora

**Affiliations:** Laboratory Medicine Department, 16582 Virgen Macarena University Hospital, Seville, Spain; Hospital Universitario Virgen Macarena, Clinical Biochemistry, Instituto de Biomedicina de Sevilla (IBIS), Consejo superior de Investigaciones Científicas (CSIC), Universidad de Loyola Andalucía - Campus de Sevilla, Seville, Spain

**Keywords:** cost-effectiveness, emergency department, overcrowding, point-of-care testing, turnaround time

## Abstract

**Objectives:**

Emergency department (ED) crowding is a quality of care and financial problem. Among its causes are long length of stay in the ED (ED LoS). One of identified causes is prolonged Turnaround Time (TAT) for complementary tests, including laboratory tests. The main aim of this study is to design and validate a cost-effective model for improving resolution of hospital emergencies at the Virgen Macarena University Hospital (VMUH) based on application of point-of-care testing (POCT) on patients classified as priority 3 (P3), according to VMUH’s triage system.

**Methods:**

P3 patients who met inclusion criteria were randomly assigned into: POCT group (laboratory tests in ED using POCT) or LAB group (laboratory tests in central laboratory). Previously, a correlation study of analytical parameters was done between both groups. Gender, age, reason for consultation, pre-intervention TAT, disposition-decision time (DDT) and ED LoS with or without imaging tests were analysed. A cost study and an extrapolation of strategy at national level were performed.

**Results:**

The correlation study proved favorable. POCT achieved a median reduction of DDT and ED LoS of 107.00 and 118.50 min respectively. This trend was maintained for non-pain related consultations and irrespective of imaging tests. Use of POCT resulted in a saving of €119.85/episode and a favorable incremental cost-effectiveness ratio (ICER) of €60.68 saved/ED LoS hour. Applying POCT to 50 % of national P3 EDs, potential savings of €284,206,701.19 were estimated.

**Conclusions:**

In conclusion, our strategy was shown to reduce DDT and, consequently, ED LoS in a cost-effective way.

## Introduction

Emergency department (ED) crowding is considered a global health problem [1], 2]. The consequences of this problem in terms of morbidity [3], mortality [4] and quality of care in EDs has received international attention, encouraging its urgent minimisation [5].

It’s not only a problem that leads to operational inefficiency in the ED, but it can also represent the collapse of the hospital in general terms [6], 7]. All this translates into an impact on the patient and healthcare staff, also affecting the cost associated with healthcare.

ED overcrowding is a major financial problem, since it is a situation of mismatch between supply and demand that is unsustainable for the healthcare system. Studies dating back almost three decades, such as that of Krochmal and Riley [8], show the large hospital costs ($6.8 million over three years) involved in this problem, using the evaluation of the increase in ED length of stay (LoS) as an indirect measure. Other studies, such as Bayley et al. [9], have found a potential loss of revenue due to ED overcrowding.

A considerable number of studies have shown that the sole cause of ED crowding cannot be found in population growth [10], [11], [12].

Factors related to ED crowding include delays caused by waiting for results of complementary studies, such as laboratory tests, which can contribute greatly to prolonging ED LoS [13] and to patient crowding in the ED. In fact, the percentage of patients attending the ED who require one or more laboratory tests is of great relevance, which is around 75 %.

Patients classified as priority 3 (P3) by the Virgen Macarena University Hospital (VMUH) triage system (Emergency Severity Index) constitute the largest group of patients attending the ED (approximately 60 % with 96,885 ED episodes attended).

P3 patients are those who present some sign of severity and this means that, despite the high probability of discharge to home, they mostly require laboratory studies to guide the decision making regarding their final destination (discharge or admission). Therefore, their waiting time in the ED is highly conditioned by the time it takes to obtain the analytical results, requiring a second consultation with the physician to close the episode definitively and, as a result, generating an increasing accumulation of patients in the ED waiting room.

One of the strategies proposed to decongest Hospital ED has been the implementation of point-of-care testing (POCT) devices in the ED, after clinical, financial and organisational evaluation [14]. POCT tests are defined as those performed at or near the point of patient care, outside the laboratory, and carried out manually, semi-automatically or automatically by non-laboratory staff. POCT devices have evolved greatly in recent years, improving their practicability and providing quality and therefore reliable analytical measurements, with results that are fully interchangeable with those that would be obtained by processing samples using central laboratory reference analysers. All these characteristics have meant that POCT systems, despite being controlled and supervised by specialised personnel from the Clinical Laboratory, can be routinely operated by non-laboratory personnel who are duly trained.

The main aims of this study were:

– Design and validate a model to improve emergencies resolution of ED based on the application of POCT technology.
– Assess the impact of POCT technology applied to a cohort of patients with P3 priority and attended in the ED of VMUH, on TurnAround Time (TAT) and ED LoS.
– Verify the usefulness of this technology as a strategy to prevent and reduce crowding in the ED.

As secondary aims, the following were proposed:

– Verify analytical quality and interchangeability of results of the POCT devices used in this study with those provided by the reference equipment located in the Clinical Biochemistry Laboratory of the VMUH (from now on, central laboratory).
– Analysis of times: between admission and first patient care (TAT A), between first patient care and medical decision time (disposition-decision time (DDT)) and ED LoS using POCT devices vs. using central laboratory equipment, in our cohort of patients.
– Assess the impact of imaging tests radiography (X-ray (XR)), ultrasound (US) and/or computed tomography (CT) on the TATs considered and on ED LoS in our cohort of patients.
– Economic evaluation of implementation of this strategy in the ED of the VMUH and extrapolation of the economic impact of this strategy to the national level.

## Materials and methods

This study was divided into two main sections:

– Analysis of TAT and ED LoS using POCT vs. using the conventional circuit. This is a prospective, cluster-randomised study.
– Economic analysis and extrapolation to national level. The following were calculated: direct, indirect (ED staff and medical supplies costs) and global costs of the POCT strategy and the incremental cost-effectiveness ratio (ICER). ICER is a mathematical tool used in health economics to analyse the cost-effectiveness of a health intervention compared to an alternative.

**Figure 1:**
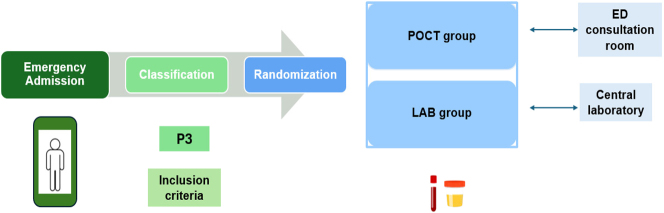
Circuit of patients attended in the VMUH ED and random assignment to study group. POCT, point-of-care testing; LAB, laboratory; ED, emergency department; P3, priority 3.

The study was carried out at VMUH between July 2023 and June 2024, with a data collection period of 3 months, from July to September 2024. The circuit that users followed in the ED is as follows (Figure 1): after passing through Emergency Admission, users classified as P3 who met the inclusion criteria of the study were randomly assigned, in the ED consultation room where they were attended and where the POCT devices were located, to one of the two groups of the study:

– Intervention group (POCT group): laboratory tests were performed at the POCT devices in the ED room where the patient was attended.
– Control group (LAB group): laboratory tests were performed in the central laboratory.

Depending on clinical suspicion, the doctor may requested laboratory tests from the profile agreed between the ED and the hospital’s Clinical Biochemistry Laboratory for this study:

– Basic metabolic panel (BMP): glucose, lactate, creatinine, urea, sodium, potassium and automatic calculation of glomerular filtration rate.
– Blood count.
– Coagulation study: activated partial thromboplastin time (APTT) and ratio, prothrombin time (PT) and ratio and International Normalized Ratio (INR), for the LAB group/INR, for the POCT group.
  – Study of acid-base and blood gas balance: venous blood gases.
  – Systematic study of urine (with sediment, in the LAB group).

In the POCT group, whole blood and/or urine were collected in, depending on the specimen: dipotassium ethylenediaminetetraacetic acid (EDTA-K_2_) tubes, blood gas syringes, capillaries and/or urine containers. In the LAB group, whole blood and/or urine were collected in, depending on the specimen: tubes with EDTA-K_2_, lithium heparin and/or sodium citrate, blood gas syringes and/or urine containers and tubes.

The test results of both groups were immediately integrated into the user’s clinical history and the physician finally decided, as in his/her usual clinical practice, the destination of each patient: discharge home, admission to the observation unit or admission to the hospitalization unit.

Subjects of the study were all those adult users who attend the VMUH ED, who were classified as P3 and who required one or more of the laboratory tests described for their care, being treated in one of the Emergency Internal Medicine consultations.

Thus, the inclusion criteria were adult patients (≥18 years) classified as P3 in the ED with a need for laboratory tests included in the analytical profile of the study and who consulted for one of the following reasons (understood as pathology, sign and/or symptom):

– Pathology of the respiratory system: decompensation of chronic obstructive pulmonary disease, flu/catarrhal symptoms, tonsillitis, pharyngitis, odynophagia, laryngitis, sinusitis, asthma attack, bronchitis, suspected pneumonia, etc.
– Genitourinary pathology, excluding pathology of the male reproductive system: dysuria, urethritis, urinary tract infection, suspected sexually transmitted disease (in women), exacerbation of chronic kidney disease, suspected renal colic or pyelonephritis, pain in the renal fossa, oliguria, etc.
– Abdominal pathology, excluding pathology of the upper hemiabdomen or biliopancreatic: gastroenteritis, gastroesophageal reflux, vomiting, suspected appendicitis, constipation, complication of inflammatory bowel disease, etc.
– Hemorrhagic pathology: gingivorrhagia, hematemesis, rectal bleeding, epistaxis, etc.
– Musculoskeletal pain: non-traumatic and moderate lumbar/cervical pain that does not require immediate analgesia, oncological pain, inguinal pain or joint pain.
– Male reproductive system pathology: orchitis, epididymitis, lump, testicular or scrotal pain, suspected sexually transmitted disease, prostatitis, etc.
– Headache/migraine.
– Cardiovascular system pathology, excluding headache/migraine: hypertensive crisis, dizziness, vertigo, edema, suspected vasovagal syncope, glycemic decompensation or paresthesia.
– Miscellaneous: asthenia, fever of unknown origin, confusion, localized cellulitis, hydroelectrolytic disorder, adenopathy, anxiety, allergic reaction, anemia or anorexia/hyporexia.

All these reasons for consultation were considered for patients classified as P3, as they allowed assessment with the analytical profile described and there was a high probability of discharge home.

The exclusion criteria were (due to greater difficulty of management in the ED): users under 18 years of age, pregnant women and/or patients with psychiatric disorders.

The withdrawal criteria were reclassification of the patient to another priority, care in the trauma, gynecological or ophthalmological circuit of the ED and/or need for analytical tests not contemplated in the study or for no analytical tests.

### Analytical methods

The POCT devices to be used and their analytical methods for the different measurements were:

– ABL90 FLEX PLUS analyzer (Radiometer; Brønshøj, Denmark): amperometric enzymatic method (glucose, lactate and creatinine), potentiometry (urea, sodium, potassium, pH and pCO_2_), optical/phosphorescence method (pO_2_) and spectrophotometry (other oximetry parameters).
– pocH-100i hematology analyzer (Sysmex; Norderstedt, Germany): hydrodynamic focus and flow cytometry for the measurement of hematimetry parameters.
– Urisys 1100 analyzer (Roche; Mannheim, Germany): photometric reflectance and refractometry method for urinalysis.
– CoaguChek^®^ XS Pro coagulometer (Roche; Mannheim, Germany): coagulometric method for electrochemical measurement of PT after coagulation activation.

The reference analysers to be used and their analytical methods for the different measurements were:

– COBAS 8000 modular analytical platform (Roche Diagnostics; Mannheim, Germany): enzymatic and photometric method (glucose), colorimetric kinetics with the Jaffé method (creatinine), kinetic and photometric method (urea) and ion-selective electrode (sodium and potassium).
– ABL 800 FLEX analyzer (Radiometer; Brønshøj, Denmark): amperometric enzymatic method (lactate), potentiometry (pH and pCO_2_), optical/phosphorescence method (pO_2_) and spectrophotometry (other oximetry parameters).
– XN-1000 hematology analyzer (Sysmex; Norderstedt, Germany): hydrodynamically focused impedance and fluorescence flow cytometry for the measurement of hematimetry parameters.
– Aution MaxTM AX-4030 analyzer (CA. Menarini Diagnostics; Florence, Italy): photometric reflectance method for urinalysis.
– Sedimax analyzer (CA. Menarini Diagnostics; Florence, Italy): refractometry method for urinalysis.
– CS-5100 coagulation analyzer (Sysmex; Norderstedt, Germany): coagulometric method for electrochemical measurements of PT and APTT after coagulation activation.

### Variables to be analyzed

– Demographic and clinical variables: gender, age and reason for consultation.
– TAT considering the existence or not of imaging tests, without considering them and according to the reason for consultation: TAT A, DDT and ED LoS.
– Costs: ED staff (medical and nursing staff), medical supplies, laboratory tests, ICER and potential cost savings with the progressive implementation of the proposed strategy at national level.

### Data collection and analysis – statistical methods

Data collection for the first section of the study was carried out retrospectively through the Electronic Digital Emergency Record and the Laboratory (LIS) and Hospital (HIS) Information Systems. For the economic analysis, data extracted from the scientific literature [15] and from Andalusia Health Services [16] were used. For the analysis of the estimated budget impact at national level, data extracted from the Specialized Care Information System (*Sistema de Información de Atención Especializada*, SIAE) of the Ministry of Health, Social Services and Equality (*Ministerio de Salud, Servicios Sociales e Igualdad*, MSSSI) were collected.

Regarding the statistical methods to be used, for the evaluation of the POCT devices, the following were used: Passing-Bablok regression, Spearman or Pearson correlation coefficient and intraclass. The distribution of the data was examined with the Shapiro-Wilk test. Continuous quantitative variables were expressed as median and interquartile range or mean ± standard deviation, while categorical variables were expressed as count and frequency of the study population. To analyse the differences between independent continuous quantitative variables, the following tests were applied, as necessary: Student’s *t*-test, Mann-Whitney U test or Kruskal–Wallis test. However, for categorical variables, the Chi-square test was used. Significance levels <5 % (p<0.05) were considered statistically significant.

The sample size should allow for the detection of differences between both groups with a power of 80 % and a significance level of 5 % using a two-tailed *t*-test and assuming a 25 % loss to follow-up. Thus, in this study, the sample size must have been from 600 consultation episodes in total, considering the inclusion of 150 due to the expected sample loss of 25 %.

## Results and discussion

### POCT vs. central laboratory correlation study

A correlation study including the parameters of interest was performed for a total of 100 samples/study group (POCT and LAB), using paired blood samples from the patients (it was not necessary to perform this study for urinalysis, as it is a semi-quantitative method).

To carry out this study, Spearman/Pearson correlation coefficients (depending on the distribution of the data) and intraclass correlation coefficients were assessed and a Passing-Bablok regression study was carried out (Table 1).

**Table 1:** Comparative study of the results obtained for the parameters indicated between POCT and central laboratory.

Test	Spearman or Pearson correlation coefficient (95 % CI)	Passing-Bablok		Intraclass correlation coefficient (R) (95 % CI)
		Slope (95 % CI)	Intersection (95 % CI)	
Creatinine, mg/dL	0.996 (0.985–0.998)	0.9560 (0.8694–1.0085)	0.017 [(−0.031)–0.093]	0.996 (0.992–0.998)
Urea, mg/dL	0.992 (0.979–0.997)	0.9331 (0.9013–1.0074)	0.399 [(−0.356)–1.032]	0.997 (0.984–0.999)
Sodium, mEq/L	0.901 (0.885–0.913)	1.0006 (0.9982–1.2101)	1.003 [(−1.742)–1.019]	0.945 (0.911–0.966)
Potassium, mEq/L	0.992 (0.974–0.995)	1.0001 (0.9078–1.0122)	−0.0840 [(−0.1223)–0.1450]	0.982 (0.874–0.993)
Glucose, mg/dL	0.996 (0.983–0.999)	0.9643 (0.9556–1.0002)	1.5714 [(−1.8469)–2.9611]	0.991 (0.887–0.994)
Hemoglobin, g/dL	0.992 (0.984–0.995)	1.0000 (0.9857–1.0111)	0.1866 [(−0.2124)–0.4784]	0.993 (0.992–0.998)
Red blood cells, ×10^6^/μL	0.993 (0.976–0.994)	1.0047 (0.9858–1.0544)	−0.1002 [(−0.1015)–0.1251]	0.990 (0.985–0.994)
Hematocrit, %	0.985 (0.981–0.989)	1.0438 (0.9859–1.1132)	−2.4816 [(−3.8479)–0.1336]	0.997 (0.975–0.998)
Mean corpuscular volume, fL	0.952 (0.887–0.976)	1.0163 (0.9441–1.1527)	−2.7522 [(−7.1523)–4.5952]	0.977 (0.956–0.981)
Mean corpuscular hemoglobin, pg	0.986 (0.966–0.990)	0.9616 (0.9128–1.1163)	0.9110 [(−0.1421)–1.996]	0.996 (0.992–0.999)
Hemoglobin concentration mean corpuscular, g/dL	0.995 (0.816–0.998)	0.4856 (0.4796–1.0074)	17.5428 [(−0.9588)–18.1365]	0.986 (0.873–0.993)
White blood cells, ×10^3^/μL	0.993 (0.982–0.996)	0.9782 (0.9253–1.1310)	0.2132 [(−0.0513)–0.3763]	0.989 (0.979–0.992)
Platelets, ×10^3^/μL	0.989 (0.973–0.991)	0.9871 (0.9562–1.0213)	0.6988 [(−10.0866)–19.9285]	0.988 (0.983–0.990)
pH blood gas analysis (venous)	0.971 (0.970–0.982)	1.0166 (1.0000–1.1033)	−0.3692 [(−0.6431)–0.0116]	0.994 (0.988–0.997)
Partial pressure of CO_2_ (pCO_2_; venous)	0.980 (0.962–0.990)	0.9945 (0.9746–1.0152)	3.7815 [(−0.0028)–4.3780]	0.989 (0.986–0.997)
Partial pressure of O_2_ (pO_2_; venous)	0.946 (0.926–0.963)	1.2619 (0.9985–1.2893)	−3.5547 [(−5.0996)–0.9541]	0.979 (0.896–0.982)
Current bicarbonate (current HCO_3_^−^; venous)	0.999 (0.981–1.000)	1.0713 (1.0000–1.1116)	−0.8003 [(−1.6869)–0.08416]	0.991 (0.983–0.995)
Standard bicarbonate (HCO_3_^−^ standard; venous)	0.986 (0.980–0.993)	1.1258 (0.9986–1.1457)	−2.5221 [(−2.8783)–0.9963]	0.988 (0.981–0.990)
Total CO_2_ content (venous)	0.982 (0.846–0.995)	1.1156 (09.714–1.1237)	−0.8014 [(−1.4545)–0.1362]	0.987 (0.979–0.991)
Base excess (in blood/current) (venous)	0.979 (0.974–0.983)	11,036 (1.0000–1.1269)	0.7490 [(−0.3559)–0.9564]	0.987 (0.963–0.989)
Excess of bases (extracellular/standard) (venous)	0.985 (0.982–0.999)	1.2327 (0.9693–1.2586)	0.8471 [(−0.2520)–0.9135]	0.995 (0.993–0.998)
O_2_ saturation (calculated) (venous)	0.976 (0.973–0.993)	0.9480 (0.9114–1.0522)	6.7366 [(−0.0117)–8.1749]	0.988 (0.874–0.996)
p50 (venous)	0.874 (0.772–0.903)	0.9874 (0.8716–1.0243)	1.1813 [(−1.1448)–2.9721]	0.941 (0.933–0.957)
INR	0.987 (0.985–0.999)	0.9744 (0.8956–1.1515)	1.0223 [(−0.0545)–1.0316]	0.992 (0.990–0.997)

95 % CI: 95 % confidence interval. INR, International Normalized Ratio.

Spearman’s or Pearson’s correlation coefficient, if applicable, was close to 1 for each test (p<0.01). No differences were observed with the Passing-Bablok regression between the two groups (95 % CI of the intercept contained the value 0 and 95 % CI of the slope contained the value 1). The intraclass correlation coefficient was also close to 1.

Therefore, a strong and positive correlation was found for all parameters evaluated between the analysers and the measurements were comparable, since, in addition, their agreement was very good (R>0.90).

### TAT analysis

The study cohort consisted of 600 patients, 300/study group. The descriptive analysis showed homogeneity in the distribution of gender, age and reasons for consultation between both groups (p>0.050) but not for the frequency of certain laboratory and/or imaging tests (p<0.050) (Table 2).

**Table 2:** Characteristics of the study cohort and univariate analysis.

Characteristics	Total (n=600)	POCT (n=300)	LAB (n=300)	p-Value
Gender (male)	299 (49.83 %)	146 (48.67 %)	153 (51.33 %)	0.624
Age	56.00 [41.00–67.00]	56.00 [40.75–67.25]	55.00 [41.00–67.00]	0.886

**Reason for consultation**				

Respiratory system disease (1)	65 (10.83 %)	32 (10.67 %)	33 (11.00 %)	1.000
Genitourinary disease (2)	128 (21.33 %)	65 (21.67 %)	63 (21.00 %)	0.921
Abdominal disease (3)	81 (13.50 %)	41 (13.67 %)	40 (13.33 %)	1.000
Haemorrhagic disease (4)	72 (12.00 %)	37 (12.33 %)	35 (11.67 %)	0.900
Musculoskeletal pain (5)	14 (2.33 %)	7 (2.33 %)	7 (2.33 %)	1.000
Male reproductive system disease (6)	24 (4.00 %)	12 (4.00 %)	12 (4.00 %)	1.000
Headache/Migraine (7)	29 (4.84 %)	14 (4.66 %)	15 (5.00 %)	1.000
Cardiovascular system disease (8)	69 (11.50 %)	33 (11.00 %)	36 (12.00 %)	0.798
Miscellaneous (9)	118 (19.67 %)	59 (19.67 %)	59 (19.67 %)	1.000

**Laboratory tests**				

Blood count	566 (94.33 %)	278 (92.67 %)	288 (96.00 %)	0.112
BMP	551 (91.83 %)	261 (87.00 %)	290 (96.67 %)	<0.050
Blood gas analysis	119 (19.83 %)	73 (24.33 %)	46 (15.33 %)	<0.050
Urinalysis	219 (36.50 %)	102 (34.00 %)	117 (39.00 %)	0.235
INR	249 (41.50 %)	61 (20.33 %)	188 (62.67 %)	<0.050

**Imaging tests (no. of patients)**	302 (50.33 %)	131 (43.67 %)	171 (56.33 %)	<0.050

XR (no. of tests)	257 (85.10 %)	109 (83.21 %)	148 (86.55 %)	<0.050
US (no. of tests)	17 (5.63 %)	7 (5.34 %)	10 (5.85 %)	0.623
CT (no. of tests)	59 (19.54 %)	24 (18.32 %)	35 (20.47 %)	0.170

Results are expressed as median and interquartile range for continuous quantitative variables and as count and frequency (percentage, %) for categorical variables. POCT, point-of-care testing; LAB, laboratory; INR, international normalized ratio; XR, X-ray; US, ultrasound; CT, computed tomography; BMP, basic metabolic panel.

As can be seen in Table 2, the use of POCT systems substantially reduced the demand for coagulation studies, although the presence of the blood gasometer in the ED office increased its use for blood gases. In addition, fewer imaging tests were performed in the POCT group overall, which would require an in-depth causal analysis.

Overall, statistically significant differences were found between the two groups for DDT and ED LoS (p<0.050) (Table 3), with a median saving of 107.00 and 118.50 min in DDT and ED LoS, respectively, for the POCT group. In contrast, no significant differences were found in the case of TAT A, which is logical given that this is a time prior to the time of the proposed POCT intervention and is therefore not affected by it. Considering the reason for consultation (Table 3), the trend was the same, except for the group numbered as 5, ‘Musculoskeletal pain’ and the group numbered as 7, ‘Headache/migraine’, as shown in Table 2, for which no significant differences were found in any of the three time periods.

**Table 3:** Analysis of TAT A, DDT and ED LoS in the overall cohort.

Global cohort											
TAT A, min			p-Value	DDT, min			p-Value	ED LoS, min			p-Value
Total	POCT	LAB		Total	POCT	LAB		Total	POCT	LAB	
40.75 [22.50–69.00]	40.00 [21.75–70.00]	41.50 [23.00–65.50]	0.912	176.50 [111.75–259.25]	122.00 [81.50–196.00]	229.00 [168.00–390.50]	<0.050	235.00 [158.00–341.00]	179.50 [120.75–252.25]	298.00 [228.50–469.25]	<0.050

According to reason for consultation									
	TAT A, min		p-Value	DDT, min		p-Value	ED LoS, min		p-Value
	POCT	LAB		POCT	LAB		POCT	LAB	
1	53.00 [30.00–93.00]	57.00 [39.00–89.00]	0.922	115.00 [90.00–192.00]	189.00 [150.00–249.00]	<0.050	186.50 [146.50–268.25]	257.00 [202.00–334.00]	<0.050
2	39.00 [19.00–65.00]	47.00 [36.00–71.00]	0.155	114.00 [79.00–181.00]	199.00 [134.00–253.00]	<0.050	162.00 [117.00–239.00]	250.00 [200.00–320.00]	<0.050
3	31.00 [16.00–56.00]	68.00 [40.00–101.00]	0.147	110.00 [80.00–205.00]	208.00 [150.00–277.00]	<0.050	150.00 [116.00–252.00]	277.00 [216.75–359.50]	<0.050
4	38.00 [25.00–56.00]	47.00 [28.00–69.00]	0.132	92.00 [66.00–168.00]	212.00 [174.00–293.00]	<0.050	143.00 [96.00–210.00]	276.00 [203.00–377.50]	<0.050
5	47.00 [37.00–68.00]	38.00 [21.00–52.00]	0.339	151.00 [133.00–190.00]	168.00 [154.00–214.00]	0.352	201.00 [186.50–239.50]	206.00 [179.00–264.50]	0.917
6	37.00 [29.00–62.00]	91.00 [33.00–123.00]	0.060	108.00 [96.00–156.00]	201.00 [173.00–235.00]	<0.050	188.00 [123.75–225.25]	277.00 [241.75–352.25]	<0.050
7	53.00 [41.00–77.00]	78.00 [42.00–88.00]	0.085	153.00 [114.00–271.00]	207.00 [200.00–281.00]	0.112	202.50 [161.00–334.50]	283.50 [241.50–310.50]	0.051
8	52.00 [17.00–88.00]	58.00 [27.00–96.00]	0.274	171.00 [106.00–219.00]	227.00 [165.50–303.00]	<0.050	225.00 [146.00–308.00]	296.00 [207.75–364.75]	<0.050
9	49.00 [25.00–71.00]	56.00 [31.00–98.00]	0.117	132.00 [72.00–173.00]	243.00 [211.50–315.00]	<0.050	180.00 [112.00–268.50]	301.00 [245.50–465,25]	<0.050

Results are expressed as median and interquartile range. TAT, turnaround time; DDT, disposition-decision time; ED LoS, emergency department length of stay; POCT, point-of-care testing; LAB, laboratory. The numbering is linked to each of the reasons for consultation considered in the study. The numbers come from Table 2.

In these two cases, DDT and ED LoS are strongly influenced by factors other than the analytical factor; that is, they are conditioned, above all, by the time that elapses until the patient’s pain is controlled, which means that POCT technology will not shorten these times significantly. In addition, the sample size for these two reasons for consultation is limited, which could also affect the limited and this could also affect the assessment of their TAT and ED LoS.

Whether or not imaging tests were performed had no impact on whether or not there were significant differences between the study groups in terms of the three time periods considered, maintaining the same trend as in the analysis of the overall cohort (Table 4).

**Table 4:** Analysis of TAT A, DDT and ED LoS in cohort of patients requiring/no requiring imaging tests.

Imaging test				
TAT, min	Total (n=302)	POCT (n=131)	LAB (n=171)	p-Value
TAT A	40.75 [24.25–86.00]	39.00 [18.50–66.50]	40.25 [32.00–94.00]	0.863
DDT	202.00 [136.50–287.00]	155.00 [106.50–219.50]	229.00 [180.00–384.50]	<0.050
ED LoS	259.00 [189.00–365.25]	208.00 [141.00–282.50]	302.00 [233.00–453.50]	<0.050

**No imaging test**				

**TAT, min**	**Total (n=298)**	**POCT (n=169)**	**LAB (n=129)**	**p-Value**

TAT A	45.50 [27.00–79.00]	42.00 [24.00–72.00]	40.25 [31.00–89.00]	0.763
DDT	153.00 [88.00–235.75]	99.00 [69.00–161.00]	227.00 [161.00–398.00]	< 0.050
ED LoS	205.00 [137.25–302.50]	157.00 [99.00–225.00]	296.00 [202.00–477.00]	< 0.050

TAT, turnaround time; DDT, disposition-decision time; ED LoS, emergency department length of stay; POCT, point-of-care testing; LAB, laboratory.

### Economic study

Costs were valued in 2019 euros according with the Andalusian Health System [16].

The cost of emergency care was assessed taking into account ED staff and medical supplies. ED staffing cost was calculated on the basis of the salaries established in the remuneration tables of the Andalusian Health System [16]. The data for the calculation of medical supplies costs were obtained from internal hospital sources and did not include pharmacy costs. In both cases, Schilling’s recommendation was followed [17], which is also included in the article by Goldstein et al. [15].

Using the above-mentioned method, the cost of 1 min of ED staffing was calculated as follows: first of all, we calculated the total cost of staffing for the ED divided by the total min per year. Staffing was considered as evenly distributed throughout the year and calculated using doctor and nursing costs only. Thus, the cost of 1 min of ED staffing was calculated to be € 28.60.

After this, following Schilling’s recommendation, the number of patients there are simultaneously in our ED was calculated by dividing the min of one year by the number of P3 emergencies per year in our hospital, resulting in an average time of 5.4 min between the arrival of two patients. So, taking into account the average ED LoS for standard practice (LAB), which is 298.00 min, this means that in our ED there were:

298.00/5.4=55 patients in the ED at any given time.

Therefore, the staffing cost per min per patient was:

(€ 28.60/min)/55 patients=€ 0.52 per min per patient.

In the same way, the medical supplies cost per min and per patient was calculated. Bearing in mind that the cost of 1 min of ED medical supplies was calculated to be € 1.99, this was equivalent to € 0.037 per patient per min.

The mean difference for each cost between both groups (POCT and LAB) was calculated and these costs were also contrasted. Finally, the total cost between both groups was also compared, taking into account both, direct cost (laboratory tests) and indirect cost (ED staff and medical supplies) (Table 5).

**Table 5:** Cost analysis of the POCT group and LAB group.

Costs, €	POCT (n=300)	LAB (n=300)	Incremental costs	p-Value
Medical supplies	7.33 (6.87–7.79)	15.60 (14.26–16.94)	−8.27 [(−9.73)–(−6.81)]	<0.050
Staff	103.04 (96.54–109.54)	219.24 (200.35–238.13)	−116.20 [(−136.68)–(−95.73)]	<0.050
Laboratory tests	5.81 (5.59–6.02)	1.18 (1.13–1.24)	4.63 (4.40–4.85)	<0.050
Total cost	116.18 (109.21–123.14)	236.02 (215.79–256.25)	−119.85 [(−141.78)–(−97.91)]	<0.050

Costs expressed in €/episode attended in the ED (mean and 95 % CI). POCT, point-of-care testing; LAB, laboratory.

The economic study revealed the following costs:

Although POCT group presented higher direct costs (laboratory tests) vs. LAB group, overall, the use of POCT resulted in a saving of €119.85/episode, as indirect costs (medical supplies and ED staff) were lower.

ICER was calculated as follows:

ICER: Total incremental cost/Median incremental ED LoS.

As it can be seen in Table 3, the median ED LoS was 179.50 min and 298.00 for POCT and LAB groups, respectively. So, the median incremental ED LoS is −118.5 min.

Therefore:

ICER: (−119.85) €/(−118.5) min=€ 1.0114/min.

Consequently, the calculation for 60 min was:

€ 1.0114/min×60 min=€ 60.68 saved/h ED LoS.

Thus, we obtained a favourable ICER at € 60.68 saved/ED LoS h.

Finally, an extrapolation of the economic impact that the implementation of the POCT strategy considered in this study would have at the national level was carried out. Using data from the MSSSI (SIAE) for 2018, the total number of ED visits in Spain was quantified at 30,372,076 with P3 emergencies accounting for approximately half (15,186,043). With the application of POCT technology to 50 % of national P3 EDs, it was estimated that potential savings of € 284,206,701.19.

In short, with the strategy presented for the improvement of ED resolution based on the application of POCT systems in patients classified as P3 in our ED, we have verified the reduction of one of the TATs that most influences the overall LoS of patients in the ED, the DDT and, consequently, in ED LoS.

This has been the case for most of the reasons for consultation considered and independently of the performance of imaging tests. It is important to highlight the great importance of focusing the strategy on P3 patients, given that these are the majority of users attended in the ED. In addition, most of these patients are likely to be discharged home after medical care, and most of them require a basic panel of laboratory tests that can be easily addressed with the available POCT devices.

In addition, it has proven to be a cost-effective strategy, since the overall costs of medical care in the overall health care costs in the intervention group were lower than in the control group.

While it is true that the robustness of our conclusions may be compromised in terms of the national extrapolation, as this is a study carried out in a single hospital centre and should therefore be validated for the centre in question when implementing the strategy presented, extrapolation of the POCT intervention to the national level indicates a huge potential cost saving from the use of POCT systems alone in 50 % of P3 patients attended.

We can therefore conclude that our approach can potentially contribute to the prevention and reduction of ED overcrowding in a cost-effective way.
